# Emergency department childhood anaphylaxis presentations in regional/remote Australia

**DOI:** 10.1111/jpc.16006

**Published:** 2022-05-04

**Authors:** Heinrich C Weber, Gaylene L Bassett, Laura K Hollingsworth, Vincent WS Gan, Samantha Rose, Jacqueline Lim, Sarah J Prior

**Affiliations:** ^1^ Tasmanian Health Service Burnie Tasmania Australia; ^2^ Tasmanian School of Medicine, Rural Clinical School, University of Tasmanian Burnie Tasmania Australia; ^3^ Tasmanian School of Medicine, University of Tasmania Burnie Tasmania Australia

**Keywords:** anaphylaxis, paediatric, regional/rural

## Abstract

**Aim:**

Explore the prevalence of childhood anaphylaxis and clinical presentation of anaphylaxis in children across two regional emergency departments over a 7‐year period.

**Methods:**

Retrospective audit of all children (0–18 years) presenting to emergency from 1 January 2010 to 31 December 2016 with anaphylaxis, defined by Australasian Society of Clinical Immunology and Allergy definitions and doctor diagnosis.

**Results:**

Seven hundred and twenty‐four patients were identified with allergic diagnosis, 60% were diagnosed with non‐anaphylaxis allergic reactions or unspecified urticaria and 40% with anaphylaxis (*n* = 286). Annual prevalence of anaphylaxis remained stable over the study period (*M* = 30.9/10 000 cases, range: 20.8–48.3/10 000). Gender distribution was equal, median age was 9.48 years (interquartile range = 4–15). Most (71%) arrived by private transport. 23% had a prior history of anaphylaxis. Food triggers (44%) were the most common cause of anaphylaxis. Insect bites/stings triggers occurred in 21%. Patients were promptly assessed (average wait time = 13 min), 16% received prior adrenaline injections. Adrenaline was administered in 26% and 20% were admitted to hospital. On discharge, 29% had a follow‐up plan, 9% received an allergy clinic referral, 6% anaphylaxis action plan, 26% adrenaline autoinjector prescriptions and allergy testing performed in 6%.

**Conclusions:**

We found a relatively low prevalence of overall childhood anaphylaxis in a regional area. The two most common causes of anaphylaxis in this population (food and bites/stings) recorded increased prevalence providing an opportunity for further study. Significant gaps in evidence‐based care of anaphylaxis were noted, demonstrating the need for improved recognition and treatment guideline implementation in regional areas.

## What is already known on this topic

1. Anaphylaxis is increasing worldwide, predominantly in the western world.
2. Increase in anaphylaxis largely due to increase in food‐related anaphylaxis, predominantly in childhood.
3. Most studies on anaphylaxis emanate from major urban centres.

## What this paper adds

1. Low prevalence of anaphylaxis in regional/rural areas and only a modest increase in food‐related anaphylaxis.
2. Bites/stings‐related anaphylaxis occur more commonly in regional/rural areas.
3. Significant gaps in childhood anaphylaxis evidence‐based care is identified in regional/rural areas.

Anaphylaxis, a severe life‐threatening allergic reaction, is on the increase globally, predominantly in the Western world.^1^ Hospital admissions for anaphylaxis have increased in the UK, USA, Canada, and Australia. In the UK, anaphylaxis hospital admissions increased by 615% over a 20‐year period, while death rates remained unchanged.^2^ Similarly, significant increases in anaphylaxis presentations have also been reported in Australia.^3^, ^4^. Furthermore, there has also been an increase in more severe presentations to emergency departments (EDs).^5^

A study of the epidemiology of anaphylaxis aids with risk assessment and appropriate management of allergy presentations. Anaphylaxis is more common in childhood with a 1 in 170 prevalence, compared to 1 in 1000 prevalence amongst adults.^6^ The increase in anaphylaxis prevalence is largely related to food‐related anaphylaxis. In an Australian study, about half (50%) of all anaphylaxis ED presentations were food‐related.^4^ In this study, the increase in food‐related anaphylaxis predominantly occurred in the 0‐4 year age group, but the greatest increase over time was noted in the 5‐14 year age group.^4^

To date, studies on anaphylaxis include mostly major urban centres. In Australia, it is reported that there is a higher prevalence of anaphylaxis in urban centres and areas of higher socio‐economic status (SES), based on prescriptions of adrenaline autoinjectors and hypoallergenic formulas.^7^ Also, food allergy presentation is increasing and the rates in urban centres were almost double compared to regional/rural areas over a 10‐year period (urban 78% compared to rural 38%).^5^ Anaphylaxis prevalence, therefore, is potentially increasing predominantly in the youngest age group (0–4 years), largely food‐related and in urban centres. However, there is a lack of published reports of anaphylaxis in regional or rural areas to assess clinical presentations and risk factors in the childhood population. In 2015, a Tasmanian study, a regional/rural area, reported on anaphylaxis presentations to paramedic ambulance staff.^8^, ^9^ These studies present potential bias as a minority of anaphylaxis episodes trigger a call to paramedics and it is likely that ED presentations will more accurately reflect the local prevalence of anaphylaxis as severe life‐threatening systemic reactions are more likely to trigger hospital presentations. Many studies have reported poor adherence to anaphylaxis treatment guidelines, such as lack of administration of adrenaline autoinjectors, poor referral patterns to allergy clinics, lack of anaphylaxis action plans and follow‐up allergy tests.^10^, ^11^ Paediatric anaphylaxis is commonly misdiagnosed in the ED and Thompson *et al*. reported that approximately half of children meeting the guideline criteria for anaphylaxis did not receive a diagnosis of anaphylaxis during an ED presentation.^12^ Also, allergy specialist referral service, a key management recommendation, may not be easily accessible to patients in regional or rural areas. Furthermore, a diagnosis of anaphylaxis for those living in regional areas is likely to result in increased morbidity due to factors such as remoteness from health‐care services.

The aim of this study is to explore the prevalence of childhood anaphylaxis in a regional area and clinical presentation of anaphylaxis in children, utilising two regional EDs, over a 7‐year period.

## Methods

### Participants

We included all children aged 0–18 years who presented to two regional ED in ‘The organisation’ with a primary diagnosis of an allergic reaction (Table 1) between 1 January 2010 and 31 December 2016. The International Classifications of Diseases (ICD) codes for urticaria were included to enable the investigators to assess the patients presenting with generalised allergic diseases for signs and symptoms of anaphylaxis. Patients who met the Australasian Society of Clinical Immunology and Allergy (ASCIA) criteria or were classified as having anaphylaxis by the treating doctor were included in this study.

**Table 1:** ICD‐10 primary diagnosis codes included in this study.

**Major category (ICD‐10 code)**
Allergic urticaria (L50.0)
Urticaria unspecified (L50.9)
Anaphylactic shock due to adverse food reaction (T78.0)
Anaphylactic shock – unspecified (T78.2)
Anaphylactic shock due to adverse effect of correct drug or medication properly administered (T88.6)

The ASCIA definition used in this study is ‘any acute onset illness with typical skin features (urticarial rash or erythema/flushing, and/or angioedema), plus involvement of respiratory and/or cardiovascular and/or persistent severe gastrointestinal symptoms; or any acute onset of hypotension or bronchospasm or upper airway obstruction where anaphylaxis is considered possible, even if typical skin features are not present’.^13^

### Method

A retrospective audit of demographic and clinical information included in digital medical records was undertaken. This included the evaluation of adherence to evidence‐based guidelines in the management of anaphylaxis, assessment of the presence of comorbidities and determining SES and remoteness classification.

### Analysis

Available data for each patient record were collated in a Microsoft Excel spreadsheet and included demographic data, initial anaphylactic reaction data, background information, clinical presentation data and discharge planning data. This data was analysed using SPSS V24.0 and included descriptive statistics and correlations were performed.

The socio‐economic disadvantage was measured using the Australian Bureau of Statistics Index of Relative Socio‐economic Disadvantage (IRSD) and Remoteness was recorded as per the Rural, Remote and Metropolitan Areas.^14^, ^15^ The IRSD is a general socio‐economic index that summarises a range of information about the economic and social conditions of households within an area and a low score on the ITSD indicates relative greater disadvantage.^14^

Ethics approval was obtained from the ‘State’ Health and Medical Human Research Ethics Committee (Reference: H0016131).

## Results

### Demographics

Seven hundred and twenty‐four children (aged 0–18 years, *M* = 7.37 years, SD = 5.59) met the criteria for review. Urticaria accounted for 60% of the participants and anaphylaxis in 40%, 77% of the latter group satisfied the ASCIA criteria for anaphylaxis or diagnosed with anaphylaxis by their treating ED doctor. Further sensitivity analyses showed no difference in outcomes between those who were diagnosed strictly according to the ASCIA definition and combined with doctor‐diagnosis of anaphylaxis. Table 2 outlines the annual allergy and anaphylaxis presentations. The annual prevalence of anaphylaxis remained stable over the study period with a mean annual prevalence of 30.9/10 000 cases (range: 20.8–48.3/10 000). Of the 724 particpants presenting with an allergy diagnosis, we observed a slight male predominance (53%) and the vast majority (709 (98%)) were residents of ‘The State’. Most (81%) arrived by private vehicle. A minority (10%) arrived by ambulance compared to 23% of those with anaphylaxis. Only 69 (9%) were admitted to hospital. One was referred to another hospital, while two left the ED unseen (Table 3).

**Table 2:** Annual prevalence of paediatric anaphylaxis presentations to ED.

Year	Allergy presentations	Anaphylaxis presentation number (ED presentation prevalence)	Food‐related anaphylaxis number(ED presentation prevalence)	Total annual paediatric ED presentations
2010	105	44 (31.0/10 000)	17 (12.0/10 000)	14 174
2011	114	41 (30.2/10 000)	16 (11.8/10 000))	13 577
2012	104	38 (28.4/10 000)	12 (8.8/10 000)	13 358
2013	115	64 (48.3/10 000)	28 (21.1/10 000)	13 254
2014	106	34 (26.6/10 000)	18 (14.1/10 000)	12 772
2015	85	39 (31.1/10 000)	17 (13.5/10 000)	12 551
2016	95	26 (20.8/10 000)	18 (14.4/10 000)	12 472
**Mean**	**103**	**41 (30.9/10 000)**	**18 (13.6/10 000)**	**13 165**

**Table 3:** Demographic data.

		*N* = 724	*N* = 286
Variable		All allergic reaction primary diagnosis	Anaphylaxis
Gender	Male	381 (53%)	147 (51%)
	Female	343 (47%)	139 (49%)
Age		*M* = 7.37 years (range: 0–18)	*M* = 9.48 years (range: 0–18)
		IQR (2–12)	IQR (4–15)
SES categories (IRSD)	1–2	513 (71%)	190 (66%)
	3–4	189 (26%)	85 (30%)
	5–10	8 (3%)	5 (4%)
Remoteness categories (RRMA)	R1 – large rural	212 (29%)	71 (25%)
	R2 – small rural	453 (63%)	197 (69%)
	R3 – other rural	45 (8%)	12 (6%)
Arrival mode	Ambulance	74 (10%)	66 (23%)
	Private vehicle	588 (81%)	204 (71%)
	Walking	59 (8%)	14 (5%)
	Other	3 (0%)	2 (1/4%)
Admission status	Admitted	69 (10%)	63 (22%)
	Departed under own care	654 (90%)	221 (77%)
	Other	3 (0%)	2 (0%)
Previous anaphylaxis		85 (12%)	66 (23%)
Asthma		131 (18%)	72 (25%)
Known food allergies		119 (16%)	81 (28%)
Allergic rhinitis		32 (4%)	17 (6%)
Eczema		122 (16%)	63 (22%)

IRSD, Index of Relative Socio‐economic Disadvantage; IQR, interquartile range; RRMA, Rural, Remote and Metropolitan Areas; SES, socio‐economic status.

The majority of participants (71%) were categorised as relatively high socio‐economic disadvantage. Most participants (63%) lived in areas classified as small rural areas. Common allergic com‐orbidities were as follows: previous anaphylactic reactions in 23%, asthma in 25%, food allergies in 28%, allergic rhinitis in 6% and eczema in 22% (Table 3).

Further, demographic data has been categorised by age and gender showing that the majority of female children presenting to ED occurs in the 12 years and older age group (49%) and that there is little variation in presentation between 2 years up to 18 years for males (Table 4).

**Table 4:** Age and gender of children presenting to ED.

		Male	Female
Variable		*N* = 147	*N* = 139
Age	<2 years	26 (18%)	15 (11%)
	2–5 years	34 (24%)	30 (22%)
	6–11 years	45 (32%)	26 (19%)
	>/= 12 years	42 (26%)	68 (49%)

### Clinical features of anaphylaxis

Skin features were the most common in clinical presentations (*n* = 266 (93%)). Respiratory features were the next most common (*n* = 175 (61%)), followed by cardiovascular (*n* = 55 (19%)) and gastrointestinal (*n* = 67 (23%)) features (Table 5). Angioedema was the most common skin presentation (*n* = 84 (32%)) and throat swelling and tightness were the most common respiratory features (*n* = 69 (39%)). The most frequently observed combination of clinical features was respiratory and skin in 169 (60%) children.

**Table 5:** Clinical features of children presenting with anaphylaxis.

Clinical features of anaphylaxis	*n*		*n*		*n*		*n*
**Respiratory**		**Cardiovascular**		**Skin**		**Gastrointestinal**	
*N* = 175 (61%)		*N* = 55 (19%)		*N* = 266 (93%)		*N* = 67 (23%)	
Difficulty/noisy breathing	34	Hypotension	6	Angioedema	84	Abdominal cramps/pain	12
Difficulty talking/hoarse voice	21	Hypertension	1	Periorbital swelling	37	Diarrhoea	3
Blisters on tongue	1	Tachycardia	7	Pruritis	60	Nausea	10
Shortness of breath	39	Pale and floppy	5	Rash	77	Vomiting	33
Wheeze	39	Pallor	9	Urticaria	79		
Chest tightness	27	Collapse	5				
Throat swelling/tightness	69	Impaired/loss of consciousness	7				
Tongue swelling	23	Mottled periphery	2				
Cough	25						
Mucous production/nasal discharge	6						
Choking	1						
Itchy throat	4						
Lip/mouth tingling	11						
Stridor	13					*N* = 286	

### Causes of anaphylaxis

Reactions occurred most frequently at home (25%), outdoors (11%), a school or childcare setting (9%) and many were not documented (36%). The major causes of anaphylaxis are as follows:

- Food‐related (*n* = 126 (44%)).
- Unidentified (*n* = 52 (18%).
- Bites/stings (*n* = 50 (21%)).
- Medications (*n* = 23 (8%)).
- Not specified (*n* = 18 (6%)).
- Idiopathic (*n* = 7 (2%)).

The most common food triggers for anaphylaxis were nuts (63%), followed by egg (21%) and cow's milk (7%). Anaphylaxis due to a bite or sting was most often caused by jack jumpers (34%), followed by bees (18%) and bull ants (8%).

**Fig. 1:**
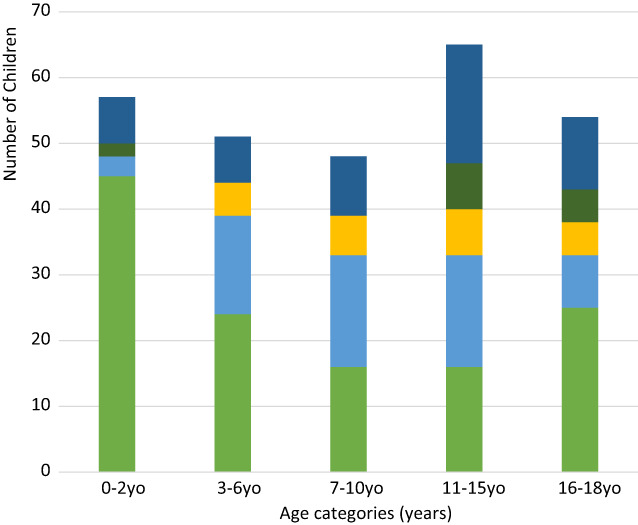
Causes of anaphylaxis by age categories. (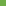) food, (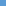) insect, (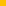) drug, (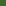) other, (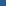) unidentified.

In children under the age of 6, food triggers were most common (64%), whereas insects (30%) and food causes (28%) were most common in the 7‐15 year age groups. Children in the 16‐18 year age group were also most affected by food causes (46%). Figure 1 shows the distribution of all categories of anaphylaxis caused by age group.

### Anaphylaxis treatment

The average wait time for children with anaphylaxis to be treated in ED was 13 min (range: 0–181 min) and 53% were treated with an antihistamine and 47% with adrenaline prior to presentation to ED. In the ED, 39% of children classified as anaphylaxis were treated with adrenaline, 45% with an antihistamine and 52% with a corticosteroid. Interestingly, there was no correlation between treatment prior to presentation and admission to a ward (*r* = 0.052, *P* = 0.390).

### Discharge

Of those presenting with anaphylaxis, one fifth (20%) required hospital admission. One child was transferred to another hospital and the reasons thereof were outside the scope of this audit. Upon discharge, just over half (52%) of children had a discharge letter sent to their GP, 29% had a follow‐up plan and 26% prescribed adrenaline autoinjector(s) for future use. Only 9% were referred to a local allergy clinic and 6% issued anaphylaxis management plan. (Table 6).

**Table 6:** Discharge process of children presenting with anaphylaxis.

Discharge mode and plan		*n*
	*N* = 286	
Discharged home		119 (42%)
Admitted to ward		57 (20%)
Transferred to another hospital		1 (0%)
Not stated		109 (38%)
Follow‐up plan		82 (29%)
Letter to GP		149 (52%)
Allergy clinic referral		26 (9%)
Anaphylaxis plan		16 (6%)
Adrenaline autoinjector		74 (26%)

Follow‐up allergy testing recorded in only 17 (6%) children and 8 (2%) had oral food challenges.

**Fig. 2:**
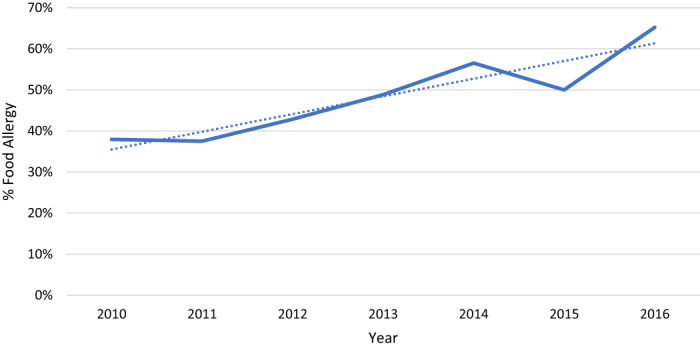
Changes in proportion of food allergy presentations.

Figure 2 shows the changes in proportion of food allergy presentations for anaphylaxis cases suggesting that over the 7‐year period between 2010 and 2016, food allergy in children has increased.

## Discussion

We describe the frequency and clinical presentations of childhood anaphylaxis in a regional/rural ED. Food allergies were the most common allergens identified, more so in the younger age group. Bites and stings were disproportionately more common triggers of anaphylaxis in our study. Although children with anaphylaxis received prompt treatment, significant gaps in evidence‐based care have been identified.

We show a relatively low prevalence of anaphylaxis, as well as food‐related anaphylaxis compared to urban areas. About 40% of children presenting to ED with allergic reactions were diagnosed with anaphylaxis, as per the ACSIA definition. This is similar to those in the local paramedic study where about a quarter had anaphylaxis,^8^ as determined by paramedics according to the paramedic diagnostic criteria, suggesting alignment of guidelines and criteria for diagnosing anaphylaxis. In the metropolitan ED study, only a tenth of allergy presentations was classified as anaphylaxis, which could be an indicator of more children with more severe allergies only accessing medical services in more remote areas.^16^ Although the results are reasonably similar in the paramedic study, it is noteworthy that only a minority (23%) of children arrived via ambulance. Interestingly, more than double of the patients who were diagnosed with anaphylaxis arrived by ambulance compared to all patients presenting with allergic reactions., reflecting the seriousness of the reactions. The lower anaphylaxis presentation in regional/rural areas with the lowest SES and more remote locations, could either be as result of a lower prevalence of anaphylaxis and allergic disease as per the hygiene hypothesis,^17^ or be reflective of poorer access to medical services.

Our cohort was older compared to those presenting with anaphylaxis to a metropolitan ED.^16^ A prior history of anaphylaxis in those presenting with anaphylaxis was more common in the metropolitan study. In the metropolitan study, more patients with anaphylaxis also had associated other atopic disorders, such as asthma and food allergies.^16^ This requires further study as it is not certain if this reflects lower prevalence of allergy‐related anaphylaxis in regional/rural areas as per the hygiene hypothesis.

The most common trigger for anaphylaxis in our study was food‐related, similar to the local ‘State’ paramedic study.^8^, ^9^ However, food‐related anaphylaxis is significantly more common in urban areas.^5^ Furthermore, our prevalence of food‐related anaphylaxis was even significantly lower than reported in rural Victoria over the same time period.^5^ We further reported a 20% increase in food‐related anaphylaxis over a 7‐year period, while metropolitan Victoria increased by 78% and 38% in rural Victoria.^5^ We report high prevalence (21%) of anaphylaxis related to insect bites and stings, similarly reported in the paramedic study,^8^, ^9^ which is significantly higher than reported in metropolitan areas (4%).^16^ The major offending insects reported in our study were jack jumpers, bees and bull ants. Reportedly, the major causes of bites/sting related anaphylaxis in ‘The State’ were jack jumper (*Myrmecia pilosula*), honeybee (*Apis mellifera*) and yellow jacket wasp (*Vespula germanica*).^18^ The only deaths of jack jumper allergy in Australia originate from ‘The State’. A further clinical concern is the long‐term risk of such anaphylaxis, as it has been reported that after an initial anaphylaxis to insect anaphylaxis, anaphylaxis as a result of re‐sting can occur up to 32 years later.^19^ This high prevalence of anaphylaxis related to bites/stings requires further study as it has implications for local preventative measures and management strategies around insect populations.

The vast majority of children with anaphylaxis in our study presented with skin manifestations, either alone or in combination with respiratory symptoms. However, Sampson *et al*. report that skin symptoms are absent in about 20% of cases of anaphylaxis.^20^ It is therefore likely that there is an element of underdiagnosis of anaphylaxis in the current study as skin presentations were almost universally present as a clinical manifestation of anaphylaxis. In the current study, children presenting with anaphylaxis were promptly assessed in ED, that is, within an average of 13 min. However, a minority received self‐administered adrenaline prior to ED presentation, which is consistent with the local ‘State’ paramedic study.^9^ This is in contrast to those presenting to an Australian metropolitan ED study where the majority of children received adrenaline autoinjector prior to presentation to hospital,^16^ and in ED, a quarter of children received adrenaline, with the vast majority (88%) receiving intramuscular adrenaline. There were no reported fatalities related to anaphylaxis during the study period, which confirms the relatively low fatality rate as reported elsewhere.^21^

Significant gaps in evidence‐based care of childhood anaphylaxis management are evident with low numbers of follow‐up plans and referrals resulting from ED admissions. This is in contrast with almost half of children with anaphylaxis at metropolitan ED were referred to an allergy clinic, while similar proportion of patients were issued with adrenaline autoinjector prescriptions. Internationally, a mean of 44% was issued with adrenaline autoinjectors and a mean of 33% was referred for allergy referrals.^22^ Potential contributors to gaps in evidence‐based care in regional and regional areas include workforce shortages, increased reliance on temporary locum workforce, access barriers for patients to specialist allergy services, lack of a clear pathway for anaphylaxis and educational materials in our institution.^22^ These gaps in evidence‐based care will require urgent study to ensure children presenting with anaphylaxis are afforded appropriate management. In Australia, the National Allergy Strategy provides a framework for addressing the challenges for children with allergic diseases.^23^ In this strategy, particular attention is also given to those living in resource‐limited or remote settings where strategies such as training other health‐care providers, other than allergists to provide allergy services and facilitating access to specialised allergy services by means of telehealth, encouraging and incentivising tertiary unit to support regional paediatric services.

A limitation of our study is the retrospective case record review which could have resulted in an element of misclassification. The life‐threatening nature of anaphylaxis would mitigate against this being a significant concern. Also, children with anaphylaxis presenting to an ED may not be representative of all children with anaphylaxis as not all patients with anaphylaxis have hospital presentations, especially in regional and rural areas access may be a factor. Furthermore, we were not able to compare rural–urban differences in Tasmania, but the ‘paramedic study’ represents childhood anaphylaxis statewide is relevant for comparison.

Conclusion

In conclusion, although childhood anaphylaxis presentations have not increased over time, we demonstrate a modest increase in food‐related anaphylaxis over the study period. We also show a very high prevalence of bites/sting related anaphylaxis locally requiring further study. Significant gaps in evidence‐based care are identified in this study, with potential life‐threatening complications.

## References

[1] Turner PJ , Campbell DE . Epidemiology of severe anaphylaxis: can we use population‐based data to understand anaphylaxis? Curr. Opin. Allergy Clin. Immunol. 2016; 16: 441–50. 27490124 10.1097/ACI.0000000000000305 PMC5325322

[2] Turner PJ , Gowland MH , Sharma V *et al*. Increase in anaphylaxis‐related hospitalizations but no increase in fatalities: an analysis of United Kingdom national anaphylaxis data, 1992‐2012. J. Allergy Clin. Immunol. 2015; 135: 956–63. 25468198 10.1016/j.jaci.2014.10.021 PMC4382330

[3] Liew WK , Williamson E , Tang ML . Anaphylaxis fatalities and admissions in Australia. J. Allergy Clin. Immunol. 2009; 123: 434–42. 19117599 10.1016/j.jaci.2008.10.049

[4] Mullins RJ , Dear KB , Tang ML . Time trends in Australian hospital anaphylaxis admissions in 1998‐1999 to 2011‐2012. J. Allergy Clin. Immunol. 2015; 136: 367–75. 26187235 10.1016/j.jaci.2015.05.009

[5] O'Loughlin R , Hiscock H . Presentations to emergency departments by children and young people with food allergy are increasing. Med. J. Aust. 2020; 213: 27–9. 32372419 10.5694/mja2.50604

[6] Boros CA , Kay D , Gold MS . Parent reported allergy and anaphylaxis in 4173 South Australian children. J. Paediatr. Child Health 2000; 36: 36–40. 10723689 10.1046/j.1440-1754.2000.00444.x

[7] Mullins RJ , Clark S , Camargo CA Jr . Socio‐economic status, geographic remoteness and childhood food allergy and anaphylaxis in Australia. Clin. Exp. Allergy 2010; 40: 1523–32. 20636400 10.1111/j.1365-2222.2010.03573.x

[8] Blackhall ML , Edwards D . Incidence and patient demographics of pre‐hospital anaphylaxis in Tasmania, Australia. Australas. J. Paramed. 2015; 12: 1–6.

[9] Edwards D , Blackhall M , Berry R . Community presentations of anaphylaxis in Tasmania: who is administering the adrenaline? Australas. J. Paramed. 2016; 13: 1–7.

[10] Huang F , Chawla K , Jarvinen KM , Nowak‐Wegrzyn A . Anaphylaxis in a new York City pediatric emergency department: triggers, treatments, and outcomes. J. Allergy Clin. Immunol. 2012; 129: 162–8. 22018905 10.1016/j.jaci.2011.09.018 PMC3246066

[11] Sidhu N , Jones S , Perry T *et al*. Evaluation of anaphylaxis management in a pediatric emergency department. Pediatr. Emerg. Care 2016; 32: 508–13. 27490724 10.1097/PEC.0000000000000864

[12] Thomson H , Seith R , Craig S . Inaccurate diagnosis of paediatric anaphylaxis in three Australian emergency departments. J. Paediatr. Child Health 2017; 53: 698–704. 28670809 10.1111/jpc.13483

[13] Guidelines A. Acute management of anaphylaxis. 2019.

[14] (ABS) ABoS . Census of population and housing socio‐economic indexes for area (SEIFA). Australia 2011;cat. no. 2033.0.55.001.

[15] Australian Institute of Health and Welfare Rural, regional and remote health: a guide to remoteness classifications. AIHW 2004; cat. no. PHE 53.

[16] Nogic C , Belousoff J , Krieser D . The diagnosis and management of children presenting with anaphylaxis to a metropolitan emergency department: a 2‐year retrospective case series. J. Paediatr. Child Health 2016; 52: 487–92. 27329902 10.1111/jpc.13173

[17] Liu AH . Revisiting the hygiene hypothesis for allergy and asthma. J. Allergy Clin. Immunol. 2015; 136: 860–5. 26449798 10.1016/j.jaci.2015.08.012

[18] Brown SG , Franks RW , Baldo BA , Heddle RJ . Prevalence, severity, and natural history of jack jumper ant venom allergy in Tasmania. J. Allergy Clin. Immunol. 2003; 111: 187–92. 12532117 10.1067/mai.2003.48

[19] Tan JW , Campbell DE . Insect allergy in children. J. Paediatr. Child Health 2013; 49: E381–7. 23586469 10.1111/jpc.12178

[20] Sampson HA , Mendelson L , Rosen JP . Fatal and near‐fatal anaphylactic reactions to food in children and adolescents. N. Engl. J. Med. 1992; 327: 380–4. 1294076 10.1056/NEJM199208063270603

[21] Yu JE , Lin RY . The epidemiology of anaphylaxis. Clin. Rev. Allergy Immunol. 2018; 54: 366–74. 26357949 10.1007/s12016-015-8503-x

[22] Burnell FJ , Keijzers G , Smith P . Review article: quality of follow‐up care for anaphylaxis in the emergency department. Emerg. Med. Australas. 2015; 27: 387–93. 26315372 10.1111/1742-6723.12458

